# Anaesthetic management and peri-operative complications in dogs undergoing cardiac electrophysiology studies and radio-frequency catheter ablations

**DOI:** 10.3389/fvets.2025.1717394

**Published:** 2025-12-04

**Authors:** Elliot Wringe, Keagan Boustead, Cristian Iovanescu, Lara Barron, Fran Downing

**Affiliations:** Davies Veterianary Specialists – (Linnaeus Veterinary Limited), Hitchin, Hertfordshire, United Kingdom

**Keywords:** veterinary anaesthesia, supraventricular tachycardia, electrophysiology, radio-frequency catheter ablation, arrhythmia < cardiovascular

## Abstract

**Background:**

The term supraventricular tachycardia (SVT) is used to describe tachydysrhythmias originating from structures at or above the atrioventricular (AV) node. While sustained SVT is uncommon in dogs, it is potentially a life-threatening arrhythmia. Electrophysiology (EP) studies and radio-frequency catheter ablation (RFCA) as a way of characterising the cause of SVT and as a definitive treatment are well described in humans and animals alike. In dogs, EP and RFCA are performed under general anaesthesia; anaesthetic management and associated complications associated with EP and RFCA in canine patients are poorly described.

**Objectives:**

This study aimed to describe anaesthetic management and evaluate peri-operative complications in dogs undergoing general anaesthesia for EP studies and RFCA for treatment of SVT at a single referral hospital.

**Methods:**

A retrospective review of the anaesthetic and hospital records of 44 dogs that underwent EP studies and RFCA at a single, referral hospital in the UK, between 2014 and 2024, was performed. Data collected included signalment, clinical findings, anaesthetic protocols, and parameters, as well as intraoperative and peri-operative complications.

**Results:**

The most common breed was the Labrador Retriever (47.7%), with a median age of 30 months (5–95 months). Anaesthetic management included premedication with a pure *μ*-opioid agonist (methadone), with or without acepromazine, induction using propofol or alfaxalone, and maintenance with isoflurane in oxygen and air via a circle rebreathing system. Intraoperative complications were recorded and included arrhythmias (97.7%), hypoventilation (84.1%), hypotension (65.1%), hypothermia (47.7%), and non-fatal cardiopulmonary arrest (CPA; 6.8%). Cardiovascular support was required in 75% of cases, including crystalloid boluses, colloids, anticholinergics, vasopressors, and inotropes. The most common arrhythmias were supraventricular tachycardia (97.7%), atrial fibrillation (13.9%), ventricular tachycardia (5.6%), ventricular fibrillation (2.8%), transient third-degree AV block (4.65%), and accelerated idioventricular rhythm (2.8%). Postoperative complications occurred in 22.7% of patients, most commonly mild gastrointestinal signs and arrhythmias. One death occurred 9 h postoperatively, resulting in an overall mortality of 2.27%.

**Discussion:**

EP studies and RFCA for SVT in dogs can be performed under general anaesthesia with a low incidence of major or catastrophic complications. Anaesthetic protocols using a *μ*-opioid agonist ± acepromazine, propofol induction, and isoflurane maintenance facilitated completion of procedures. Although intraoperative arrhythmias, hypotension, and hypoventilation were common, these events were manageable. Severe complications and peri-operative mortality were uncommon, and overall outcomes were favourable. Careful anaesthetic planning, consideration of the severity of underlying cardiac disease, and preparedness for cardiopulmonary emergencies are recommended to optimise patient management and outcomes.

## 1 Introduction

Supraventricular tachycardias (SVT) are pathological, typically narrow complex, rapid arrhythmias, originating from structures at or above the atrioventricular (AV) node. Causes include atrioventricular accessory pathways (APs), focal atrial tachycardia (FAT), atrial fibrillation, and atrial flutter (1, 2).

Orthodromic atrioventricular reciprocating tachycardia (OAVRT; secondary to the presence of an AP) and focal atrial tachycardia (FAT) are two common causes of supraventricular tachycardia (SVT) in dogs, with OAVRT accounting for approximately 50–70% of cases and FAT representing around 10–20% (3). OAVRT arises from retrograde conduction through congenital accessory pathways that bypass the normal electrical insulation of the annulus fibrosus following ventricular depolarisation; these are typically right sided (most often in a right posteroseptal or posterior location) and have potentially been linked to tricuspid valve dysplasia (4–8). This retrograde conduction can trigger premature atrial depolarisation, creating a macro re-entrant circuit and causing an SVT exceeding 250 beats per minute (9). FAT originates from a single focal source within the atrium, producing a narrow-complex tachycardia with rates typically between 210 and 340 bpm (2, 3, 10). In dogs, FAT ectopic foci most commonly localise to the right atrium (63%), near structures such as the crista terminalis, tricuspid annulus, coronary sinus, and pulmonary vein ostia (2, 3). Early clinical signs of both arrhythmias are often vague and transient, including lethargy, exercise intolerance, syncope, and gastrointestinal symptoms, but may lead to congestive heart failure (2, 8).

Sustained or episodic SVT can lead to tachycardia-induced cardiomyopathy (TICM), resulting in a dilated cardiomyopathy (DCM) phenotype on echocardiography (9, 11) and associated clinical signs (exercise intolerance, dyspnoea/tachypnoea, and cough). TICM results from myocardial energy depletion and ischaemic injury due to increased oxygen demand, elevated left ventricular end diastolic pressure, and reduced coronary perfusion during shortened diastole (12).

Management of SVT involves rate control with anti-arrhythmic agents and treatment of TICM and/or congestive heart failure. Long-term management can be achieved via radiofrequency catheter ablation (RFCA) of the accessory pathway(s) or ectopic foci following electrophysiology (EP) and conduction mapping (7). In dogs, this involves fluoroscopically guided, transvenous insertion of multipolar catheter electrodes into the cardiac chambers to locate and thermally ablate the pathological conduction pathways. Reported success rates for RFCA for SVT are between 90 and 96% in humans and 91–98.8% in dogs (4, 8, 13–15). In canine patients, RFCA for management of SVT is performed in only a handful of specialist centres globally.

In adult humans, EP studies are typically performed under local anaesthesia with or without sedation, while in paediatric patients, general anaesthesia (GA) is usually required. General anaesthesia is required in all veterinary patients undergoing EP and RFCA to ensure patient and personnel safety. While EP and RFCA are well-documented in veterinary literature, to the authors’ knowledge, there is little published information about the anaesthetic management and peri-operative complications associated with these procedures.

The primary objective of this study is to describe the anaesthetic management and incidence of peri-operative complications associated with EP and RFCA at a single veterinary centre. A secondary objective was to evaluate any potential associations between anaesthetic management practices and intraoperative complications.

## 2 Materials and methods

The study was approved by the RCVS Ethical Review Panel. Anaesthetic and hospitalisation records of dogs that underwent GA for EP study and RFCA procedures following diagnosis of SVT by a board-certified veterinary cardiologist, between the years of 2014 and 2024, were collated at Davies Veterinary Specialists (Hertfordshire, United Kingdom). Population inclusion was via search words “electrophysiological study,” “electrophysiology,” and “ablation” on the hospital recording system Animana (Idexx). Inclusion criteria were any dog that underwent an electrophysiology study and RFC ablation procedure between the years 2014 and 2024. Exclusion criteria included missing records (anaesthetic and/or hospitalisation) and patients having undergone previous interventional cardiac surgery.

Patient data recorded included results of the pre-anaesthetic clinical assessment, including temperature, pulse rate, respiratory rate, signalment (breed, gender, age, weight, and body condition score), pre-existing cardiac disease, presence of non-cardiac co-morbidities, and long-term or recent medications.

Data recorded about the anaesthetic management included American Society of Anesthesiologists (ASA) physical status classification (16); premedication agents used, including doses and routes of administration; induction agents and doses; maintenance agents and carrier gases; intraoperative monitoring parameters (end-tidal CO_2_ [ETCO_2_], temperature, blood pressure [43 via invasive monitoring and 1 via non-invasive monitoring], heart rate, respiratory rate, and haemoglobin oxygen saturation); duration of anaesthesia and procedure. Intraoperative complications and interventions were recorded, including arrhythmias, hypothermia, hypoventilation, hypotension, use of positive pressure ventilation (either volume or pressure-controlled ventilation), and pharmacological intervention for the provision of analgesia and/or cardiovascular support. Intraoperative hypotension was defined as mean arterial pressure <60 mmHg for two consecutive readings, taken at 5-min intervals via either non-invasive oscillometric devices or invasively following placement of an arterial cannula. Mild hypercapnoea was defined as an end tidal CO_2_ (ETCO₂) > 45 mmHg for ≥10 min; marked hypercapnoea as ETCO_2_ > 60 mmHg for the same duration. Hypothermia was defined as oesophageal temperature <36.5 °C. Hypoxaemia was defined as an oxygen saturation (SpO_2_) < 95% for ≥10 min. Postoperative respiratory rate, heart rate, and the presence of arrhythmias were recorded every 4 h and temperature every 12 h until discharge. Patients undergoing procedures after 2022 received constant ECG monitoring due to the availability of a telemetric monitoring system (BeneVision TM80; Mindray Animal Medical, China). All postoperative complications documented between recovery and discharge were recorded. Results were compiled using Microsoft Excel (Microsoft Office Suite, version 16.84).

Anaesthetic management plans were determined by the attending veterinary anaesthetist, all of whom were either diplomates of the European College of Veterinary Anaesthesia and Analgesia (ECVAA), ECVAA residency-trained, or ECVAA residents working under the direct supervision of an ECVAA diplomate. All cardiac interventional procedures were performed by a board-certified veterinary cardiologist with or without the assistance of a human cardiologist.

## 3 Statistical analysis

Statistical analysis was performed in R, version 4.4.3, using RStudio/2024.12.1 + 563. Categorical variables were summarised by count and proportion. Numerical data were inspected for normality by histogram plots and Shapiro–Wilk tests. Normally distributed data were reported as mean ± standard deviation, while non-normal data were reported as median (range).

The number of dogs experiencing specific arrhythmia before the EP study was compared to the number of dogs experiencing the same arrhythmia under general anaesthesia. For these comparisons, McNemar’s exact test was used. The odds ratio and Bonferroni-adjusted 95% confidence intervals and *p*-values were reported. As zero counts in the VF and 3^rd^ degree AV block groups resulted in infinite confidence intervals and odds ratios, the exact binomial test was performed for these arrhythmias, and the probabilities of developing these arrhythmias were reported rather than odds ratios. *Post-hoc* power of each test was also calculated. During this analysis, the following arrhythmias were grouped together: atrial fibrillation and atrial flutter (grouped as AFib/Flut); ventricular tachycardia, accelerated idioventricular rhythm, unspecified wide complex tachycardia (grouped as WCT). The remaining rhythms were analysed without grouping. These were as follows: OAVRT, ventricular fibrillation (VF), FAT, and 1^st^ and 3^rd^ degree atrioventricular (AV) block. Results are shown in Table 1.

**Table 1:** Proportion of dogs with specific arrhythmias recorded pre-procedurally and during general anaesthesia undergoing electrophysiological study.

Arrhythmia	Percentage (n); recorded preprocedure	Percentage (n); recorded under general anaesthesia	Odds ratio or probability* (Adjusted 95% CI)	Adjusted *p*-value	Power (%)
OAVRT	63.64% (28)	86.36% (38)	3.25 (0.69–25.79)	0.39	53.4
Afib/Aflut	6.98% (3)	13.63% (6)	4 (0.20–1,596)	1	10
Focal atrial tachycardia	6.98% (3)	6.82% (3)	1 (0.00–638.50)	1	0
Wide complex tachycardia	4.65% (2)	6.82% (3)	1.5 (0.08–4.55)	1	15
Ventricular tachycardia	0% (0)	2.27% (1)	1* (0.03–1)	1	0
Accelerated junctional rhythm	4.65% (2)	0% (0)	0 (0–13.14)	1	1.4
First-degree AV block	2.33% (1)	0% (0)	0 (0–319)	1	0
Third-degree AV block	0% (0)	4.54% (2)	1* (0.16–1)	1	1.3

For these arrhythmias, the odds ratio (odds of arrhythmia under general anaesthesia vs. preprocedure) or probability of arrhythmia developing under GA(*) with Bonferroni-adjusted 95% confidence intervals, *p*-values, and model power are reported. (OAVRT, orthodromic atrioventricular reciprocating tachycardia; AFib/AFlut, atrial fibrillation/atrial flutter; AV, atrioventricular block).

An exploratory analysis was performed to identify variables related to the intraoperative complications: hypotension, hypothermia, and marked intraoperative hypercapnia. This analysis was done in a stepwise approach. First, the data was explored for missing values. These values were imputed as the mean if numeric, or into a specific group with the use of classification and regression trees.

Next, to narrow down potential influential variables for each outcome variable, conditional permutation variable importance was computed using a conditional inference forest with repeated (5 times) 10-fold cross-validation. For this analysis, the number of trees was set at 500, and the number of variables considered at each node was the square root of the number of predictors. Variables included in the models are listed in Table 2. Variables with a mean importance score of more than zero were further analysed by best subset logistic regression. The top 3 models were selected based on the lowest Corrected Akaike Information Criterion (AICc), the odds ratios, Bonferroni-adjusted 95% confidence intervals, and *p*-values of the variables in the 3 best logistic regression models were extracted. Alpha was set at 0.05% for all analyses. *Post-hoc* power calculations for each predictor in each logistic regression model were performed.

**Table 2:** Summary of all predictor variables included in the conditional inference forest models for dogs undergoing general anaesthesia for EP and RFCA procedures.

Variable	Type	Hypotension	Hypothermia	Marked hypercapnia	Missing (n)
Age (months)	N	✓	✓	✓	0
Weight (kg)	N	✓	✓	✓	2
Sex	C	✓	✓	✓	0
Breed (Labrador vs. other)	B	✓	✓	✓	0
Normal echocardiogram	B	✓	✓	✓	0
Amiodarone (preprocedural)	B	✓	✓	✓	0
Sotalol (preprocedural)	B	✓	✓	✓	0
Mexiletine (preprocedural)	B	✓	✓	✓	0
Pimobendan (preprocedural)	B	✓	✓	✓	0
Diltiazem (preprocedural)	B	✓	✓	✓	0
Diuretics (preprocedural)	B	✓	✓	✓	0
Benazepril (preprocedural)	B	✓	✓	✓	0
Acepromazine (premedication)	B	✓	✓	✓	0
Anaesthesia time	N	✓	✓	✓	0
Fentanyl CRI	B	✓	✓	✓	0
Benzodiazepine (co-induction)	B	✓	✓	✓	1
Fentanyl (co-induction)	B	✓	✓	✓	1
Intraoperative paracetamol	B	✓	✓	✓	0
Intraoperative methadone	B	✓	✓	✓	0
Fentanyl bolus (intraoperative)	B	✓	✓	✓	0
Neuromuscular block	B	✓	✓	✓	0
Intraoperative SVT	B	✓	✓	✓	0
Intraoperative atrial fibrillation	B	✓	✓	✓	0
Wide complex tachycardia	B	✓	✓	✓	1
Focal atrial tachycardia	B	✓	✓	✓	0
Third-degree AV block	B	✓	✓	✓	0
Highest heart rate	C	✓	✓	✓	1
Mechanical ventilation	B	✓	✓	✓	0
Lowest heart rate	N	✓	✓	✓	1
Intraoperative hypothermia	B	✓	Outcome	✓	0
Intraoperative hypotension	B	Outcome	✓	✓	1
Intraoperative amiodarone	B	✓	✓	✓	0
Intraoperative Ca2 + channel blockers	B	✓	✓	✓	0
Intraoperative lidocaine	B	✓	✓	✓	0
ETCO₂ > 45 mmHg	B	✓	✓	✓	0
ETCO₂ > 65 mmHg	B	✓	✓	Outcome	0
Trazodone (preprocedural)	B	✓	✓	✓	0
Maropitant (preprocedural)	B	✓	✓	✓	0
Cardiopulmonary arrest	B	X	✓	✓	0
Anticholinergic	B	X	✓	✓	0
Dobutamine	B	X	✓	✓	0
Noradrenaline	B	X	✓	✓	0
Fluid bolus	B	X	✓	✓	0
Defibrillation	B	X	✓	✓	0

Specifying data type, relevance to each outcome (hypotension, hypothermia, and marked hypercapnia), and the number of missing observations. “✓” indicates that the variable was considered as a possible predictor for the respective outcome, “Outcome” denotes that the variable represents the actual outcome rather than a predictor, and “X” indicates exclusion from the model for that outcome. (CRI, Constant Rate Infusion; SVT, Supraventricular Tachycardia; A-fib, Atrial Fibrillation; AV, Atrioventricular; ETCO₂, End-Tidal Carbon Dioxide; N, numeric; C, categorical; B, binary; ✓, included in model; X, not included; Outcome, dependent variable).

## 4 Results

A total of 47 EP studies and RFCA were performed over the 10-year period. Three dogs were excluded from the study; 1 case was excluded due to previous cardiac interventional surgery, and 2 for missing/incomplete hospitalisation or anaesthetic records. Therefore, statistical analysis was performed on 44 cases. With regards to the data set, a total of 11 missing values (out of a total of 1980 data points) were found in the data set. One variable (ASA class) had three missing values, so it was excluded from further analysis. The remaining 8 missing values were randomly distributed amongst the variables. Of these, 39 cases were suspected to have OAVRT and 3 FAT. One case had empirical ablation due to presumptive atrial flutter. One case did not receive an ablation due to no abnormal conductance or inducible arrhythmia being discovered or elicited during the procedure.

The study population was made up of 23 male entire, 11 female entire, 6 female neutered, and 4 male neutered dogs, with the most commonly represented breed being the Labrador Retriever (47.73%, *n* = 21). All breeds are summarised in Table 3. Median age was 30 months (5–95 months); median body condition score was 5 (range 2–8) (17); mean body weight (± SD; kg) was 27.56 ± 8.21; median ASA score was 3 (2–4).

**Table 3:** Distribution of dog breeds included in the study population undergoing GA for EP and RFCA procedures.

Breed	% of study population (count)
Labrador Retriever	47.73% *(n = 21)*
Golder Retriever	15.9% *(n = 7)*
Cocker Spaniel	6.82% *(n = 3)*
Cross Breed	6.82% *(n = 3)*
Rottweiler	4.55% *(n = 2)*
Basset Hound	4.55% *(n = 2)*
English Bulldog	4.55% *(n = 2)*
Deer Hound	2.27% *(n = 1)*
Border Collie	2.27% *(n = 1)*
English Springer Spaniel	2.27% *(n = 1)*
Staffordshire Bull Terrier	2.27% *(n = 1)*

For each breed, the percentage of the total study population is shown alongside the count (n).

Patients were presented for EP study due to evidence of SVT on 24-h ECG Holter analysis. Pre-procedural ECG findings performed the day of, or the day before the procedure documented: suspected OAVRT in 28 dogs (65.12%), sinus rhythm with or without pre-excitation (23.23%, *n* = 10), FAT (6.98%, *n* = 3), accelerated junctional rhythm (4.65%, *n* = 2), wide complex tachycardia (4.65%, *n* = 2), atrial flutter (4.65%, *n* = 2), atrial fibrillation, (2.33%, *n* = 1), and first degree AV block (2.33%, *n* = 1). Some dogs displayed more than one of the aforementioned arrhythmias during assessment. Twelve patients (27.27%) had no abnormalities on pre-procedural ECG. Ten patients (23.23%) had no abnormalities on pre-procedural echocardiogram, with the remainder having evidence of either chamber enlargement with or without decreased systolic function, and or valvular regurgitation. The severity and form of structural disease were not quantified.

Oral medications at the time of the procedure are summarised in Table 4. Premedication included methadone, acepromazine, and alfaxalone (Table 5). The route of premedication was intravenous in 52.3% of patients (*n* = 23) and intramuscular in 47.7% (*n* = 21). All patients received cefuroxime, 22 mg kg^−1^ intravenously at 90-min intervals throughout the duration of GA.

**Table 4:** Oral medications administered to dogs presenting for general anaesthesia for EP and RFCA procedures at the time of the procedure.

Medication	Percentage (count)
Pimobendan	59.09% *(n = 26)*
Sotalol	54.54% *(n = 24)*
Diltiazem	50.00% *(n = 22)*
Mexilitine	38.63% *(n = 17)*
Furosemide	15.91% *(n = 7)*
Amiodarone	13.63% *(n = 6)*
Benazepril	9.09% *(n = 4)*
Spironolactone	9.09% *(n = 4)*
Trazodone	4.56% *(n = 2)*
Maropitant	4.56% *(n = 2)*
Torasemide	2.27% *(n = 1)*
Digoxin	2.27% *(n = 1)*
Gabapentin	2.27% *(n = 1)*
Omeprazole	2.27% *(n = 1)*
Metronidazole	2.27% *(n = 1)*
Mirtazapine	2.27% *(n = 1)*

Values are reported as the percentage of dogs receiving each medication and count (n).

**Table 5:** Drugs administered to dogs presenting for EP and RFCA for premedication and induction of anaesthesia, including the median dose (range), route of administration, and the number of dogs that received each medication.

Drug	Dose (Median and range)	Route of administration	Number of dogs (%)
Premedication			
Methadone	0.25 mg kg^−1^ (0.1–0.3)	IV, IM	44 (*100%*)
Acepromazine	0.01 mg kg^−1^ (0.003–0.02)	IV, IM	23 (*52.27%*)
Alfaxalone	0.75 mg kg^−1^	IM	1 (*2.27%*)
Primary induction agent			
Propofol	*3.26 mg kg^−1^ (0.66–6.25)	IV	43 (*97.72%*)
Alfaxalone	1.8 mg kg^1^	IV	1 (*2.27%*)
Co-induction agents			
Midazolam	0.2 mg kg^−1^ (0.2–0.3)	IV	25 (*56.82%*)
Fentanyl	1.5 mcg kg^−1^ (1–6)	IV	11 (*25%*)
Diazepam	0.2 mg kg^−1^ (0.2–0.3)	IV	5 (*11.63%*)
Lidocaine	1 mg kg^−1^	IV	1 (*2.27%*)

The induction agents are divided into the primary induction agent (with all dogs receiving either propofol or alfaxalone) and co-induction agents, which are given in addition to a primary agent. * Doses expressed as median and range, as propofol administration was titrated to clinical effect rather than predetermined.

Anaesthesia was maintained using isoflurane carried in an air/oxygen mix using a circle re-breathing system in all cases. Average anaesthetic time was 198.07 ± 60.13 min, with surgical time averaging 151.31 ± 60.58 min. Intermittent positive pressure ventilation (IPPV) was used in 38 patients (86.36%): 24 via volume-controlled, pressure-limited ventilation; 4 via pressure-controlled ventilation. In 10 patients, the method of delivering IPPV was not recorded. The mean maximum inflation pressure was recorded at 14.5 cm H20 and 13.5 cm H2O for each method, respectively. Fentanyl was administered as a bolus in 21 patients (47.73%) at a median dose of 2 mcg kg^−1^ (1–7.5 mcg kg^−1^); fentanyl constant rate infusions were used in 3 dogs (6.82%) at mean dose of 4.3 mcg kg hour^−1^ (3–5 mcg kg hour^−1^) and an alfentanil constant rate infusion was used in 2 dogs (4.54%) at a rate of 1 mcg kg min^−1^ in both cases. Additional IM methadone was given to 15 patients (34.1%) prior to recovery at a median dose of 0.1 mg kg^−1^ (0.1–0.3 mg kg^−1^). Intravenous paracetamol at a median dose of 15 mg kg^−1^ (10–15 mg kg^−1^) was given to 17 dogs (38.64%).

Intraoperative complications were as follows: arrhythmia (97.7%, *n* = 43), hypoventilation (84.1%) [classified as mild (84.1%, *n* = 37) or marked hypercapnoea (25%, *n* = 25)], hypotension (65.12%, *n* = 28), hypothermia (47.73%, *n* = 21), and cardiopulmonary arrest (6.82%, *n* = 3).

Intraoperative arrhythmias were classified by the attending anaesthetist as OAVRT (86.36%, *n* = 38), atrial fibrillation (13.89%, *n* = 6), ventricular tachycardia (5.56%, *n* = 2), focal atrial tachycardia (5.56%, *n* = 2), transient 3rd degree AV block (5.56%, *n* = 2), and single cases of ventricular fibrillation and accelerated idioventricular rhythm (AIVR). 3rd degree AV block occurred in two cases after ablation attempts; however, it resolved without intervention, and pacemaker implantation was not required. Intraoperative cardiovascular and antiarrhythmic interventions are summarised in Table 6.

**Table 6:** Intraoperative cardiovascular support interventions administered to dogs under anaesthesia during the EP study and RFC ablation.

Intervention	No. of cases (%)	Dose / volume / route of administration	Reason for administration
Isotonic crystalloid bolus (Hartmann’s solution)	16 (36.36%)	8.74 mL kg^−1^ ± 5.12 IV	75% (*n = 12*) Recorded as hypotensive at the point of administration
Synthetic colloid bolus (Geloplasma®)	3 (6.81%)	4 ml kg^−1^ (± 2) IV	All recorded hypotensive at the point of administration
Atropine	8 (18.18%)	0.019 mg^−1^ kg^−1^ (0.005–0.04) IV	Arrhythmia induction (*n = 4*) Bradycardia (*n = 1*) Post-ablation testing (*n = 1*) CPA (*n = 1*) Reason not recorded (*n = 1*)
Glycopyrrolate	1 (2.27%)	0.005 mg kg^−1^ IV	Arrhythmia induction (*n = 1*)
Adrenaline	1 (2.27%)	0.01 mg kg^−1^ IV	CPA
Noradrenaline	2 (4.54%)	0.04–0.15 mcg kg^−1^ min^−1^ IV	50% (*n = 1*) recorded hypotensive at the point of administration
Dobutamine	3 (6.81%)	3.6 mcg kg^−1^ min^−1^ (3–4) IV	All recorded hypotensive at the point of administration
Amiodarone	2 (4.54%)	Not specified	Atrial fibrillation (*n = 1*) Refractory SVT (*n = 1*)
Diltiazem	3 (6.82%)	Not specified	SVT
Lidocaine	3 (6.82%)	Not specified	SVT (*n = 2*) Refractory ventricular fibrillation (*n = 1*)
Verapamil	1 (2.27%)	0.025 mg kg^−1^	Atrial fibrillation

Values represent the number of dogs (n) receiving each intervention and the corresponding percentage of the total population (%). The dose or volume administered is shown where available, and the reason for administration reflects the clinical context at the time of use. Doses are expressed as mean and range (CPA, Cardiopulmonary arrest; SVT, Supraventricular tachycardia).

The results of the exploratory analysis are reported in Table 7. For all evaluated logistic regression models, there were no significant predictors for the outcomes hypotension, hypothermia, and marked hypercapnoea (*p* > 0.05).

**Table 7:** Predictors of intraoperative hypotension, hypothermia, and marked hypercapnia in dogs undergoing general anaesthesia for EP and RFCA.

Outcome variable	Top predictors identified by conditional inference forest	Top logistic models: based on AICc	Model predictors	Odds Ratios (Adjusted 95% CI) for model predictors	Adjusted *p*-value for model predictors	Power (%) of the model for each predictor
Hypotension	Intraoperative paracetamol ACP premedication Preoperative Mexiletine Age	1	Intraoperative paracetamol	3.73 (0.71–29.0)	0.46	44.2
		2	ACP premedication	2.24 (0.45–12.30)	1	17.8
			Intraoperative paracetamol	3.87 (0.71–31.0)	0.44	45.3
		3	Intercept	-	-	-
Hypothermia	Preoperative Mexiletine Body weight Intraoperative Paracetamol Sex	1	Preoperative Mexiletine	3.12 (0.65–17.20)	0.55	44.5
		2	Intraoperative Paracetamol	3.12 (0.65–17.20)	0.55	44.5
		3	Body weight	1.06 (0.96–1.19)	1	3
			Preoperative Mexiletine	3.42 (0.68–20.50)	0.46	17.4
Marked hypercapnia	Sex Age	1	Age	0.97 (0.90–1.01)	0.81	39
		2	Intercept	-	-	-
		3	Female	0.22 (0.02–1.13)	0.38	2.65 (vs. Male)
			Female neutered	0.90 (0.01–22.20)	1	0.45 (vs. Female)
			Male	1.97 (0.26–26.6)	1	3 (vs. Female)
			Male neutered	1.50 (0.02–43.1)	1	1.5 (vs. Female)

Top predictors identified using conditional inference forest analysis and corresponding logistic regression models based on corrected Akaike information criterion (AICc). Reported values include odds ratios (OR) with Bonferroni-adjusted 95% confidence intervals (CI), adjusted *p*-values, and statistical power (%) for each model predictor (ACP, acepromazine; AICc, corrected Akaike information criterion; CI, confidence interval).

The results of the comparison between the number of dogs experiencing a specific pre-procedural arrhythmia vs. the same arrhythmia under general anaesthesia can be found in Table 1. No significant differences were found for OAVRT, FAT, wide-complex tachycardia (WCT), ventricular fibrillation (VF), first- and third-degree AV block, atrial fibrillation/atrial flutter, and accelerated junctional rhythm (*p* > 0.05).

Three patients (6.81%) underwent electrical cardioversion for intraoperative atrial fibrillation (AF). The breeds included a Labrador Retriever, a Golden Retriever, and a Rottweiler. The number of shocks delivered was 6, 2, and 2, respectively, with a mean energy of 78.75 J. Three dogs went into cardiopulmonary arrest (CPA) under general anaesthesia: two Labrador Retrievers and one Cocker Spaniel. One case developed sudden, severe bradycardia following induction and received atropine at 0.04 mg kg^−1^, chest compressions, and IPPV. Return of spontaneous circulation (ROSC) was achieved, and the RFCA was performed to completion. One dog experienced prolonged, refractory VF following endocardial pacing and was successfully resuscitated following sequential defibrillation. The procedure was aborted, and the dog recovered uneventfully. One dog experienced sustained SVT, which precipitated cardiopulmonary arrest; ROSC was achieved following defibrillation, intravenous adrenaline, chest compressions, and IPPV. The procedure was aborted, and the dog recovered with only mild swelling/haemorrhage at cannulation sites. Identification of the arrhythmia from the anaesthetic monitoring record during the CPA was unclear in this instance.

Postoperative complications were recorded in 22.73% (*n* = 10) of cases, with the average time from extubation to discharge being 24.42 h (15–76 h). The most common were gastrointestinal signs (vomiting/regurgitation) in 3 (6.82%) and intermittent accelerated idioventricular rhythm in 2 dogs (4.54%). Other complications, each reported in a single case (2.27%), included atrial fibrillation, azotaemia (creatinine 326 μmol/L, BUN 16.7 mmol/L), bleeding at the sites of cannulation, hypothermia, intermittent ventricular tachycardia, and postoperative fatal arrhythmia.

## 5 Discussion

This is the first veterinary study solely investigating anaesthetic management and occurrence of peri-operative complications in dogs undergoing electrophysiology studies with or without successful radio-catheter ablation at a single referral facility over a 10-year period.

Labrador Retrievers were the most common breed represented, accounting for 47.73% (21 of 44) of the cohort, followed by Golden Retrievers at 15.9% (7 of 44). This distribution aligns with previous studies reporting 47–61% Labrador Retriever representation in EP investigations (6, 8, 18). The reason for the high prevalence of accessory pathways in these breeds is unknown; however, it may be linked to a single recessive allele associated with tricuspid valve dysplasia (TVD), a condition with a genetic basis in Labrador Retrievers (19).

Pre-procedural oral medications were administered frequently for reasons including optimising cardiac function, arrhythmia management, managing gastrointestinal disease, and anxiolysis/sedation; however, due to the retrospective nature of the study, it was not possible to accurately differentiate between patients who were receiving medication at the time of the procedure and those who had discontinued them prior. Other studies suggest that antiarrhythmic medications should ideally be discontinued at least five half-lives before EP study, to prevent conduction suppression, which may interfere with the identification of pathological pathways or falsely imitate ablation success (3, 8). Pimobendan has been proposed to exert positive inotropic effects and enhance cardiac output via calcium sensitisation and reduce afterload via vasodilation in dogs and may therefore have potential benefit during EP and RFCA procedures, particularly in patients with impaired systolic function (20, 21). Vasodilatory agents, including ACE inhibitors, were administered based on clinical presentation or clinician discretion, given their potential to exacerbate hypotension during general anaesthesia (22). The influence of preoperative oral medications on anaesthetic management and peri-operative complications remains undefined and warrants further investigation.

Premedication selection in patients undergoing EP and RFCA is influenced by multiple factors, including the need for analgesia and sedation/anxiolysis. Agents that provide analgesia may be beneficial to address discomfort from vascular access port placement and the ablation procedure itself. In humans, radiofrequency ablation has been associated with moderate to severe pain in 60% of patients within the first 24 h post-procedure (23, 24). In the current study, all patients received methadone, hence preventing any comparison to alternative opioid or non-opioid-based pre-medication protocols. Sedation and anxiolysis are desirable properties of pre-anaesthetic medication, facilitating venous cannulation, surgical site preparation, and potentially reducing the doses required for induction and maintenance of anaesthesia. Lower anaesthetic maintenance requirements can decrease the frequency and severity of dose-dependent side effects, including peripheral vasodilation, while preserving myocardial contractility, cardiac reflexes, and regional blood flow autoregulation (25, 26); this may be particularly important in patients with compromised cardiac function. In patients undergoing EP/RFCA, agents that significantly suppress cardiac conductivity may interfere with the identification of accessory pathways or ectopic foci.

Methadone was the most common drug administered for premedication, with or without ACP. Methadone, a full *μ*-opioid receptor agonist, is widely used in veterinary anaesthesia for its potent analgesic and mild sedative properties. It is often used in conjunction with acepromazine, a phenothiazine sedative with dopaminergic, serotonergic, adrenergic, and histaminic antagonistic activity (27). At low doses, this combination provides mild to moderate sedation with relatively stable cardiovascular effects, although opioid-induced bradycardia and *α*-adrenergic–mediated vasodilation may occur (28).

ACP has been reported to exert protective effects against catecholamine-induced arrhythmias while providing sedation and reducing induction agent requirements (29, 30). In patients with impaired systolic function, such as those with TICM or compromised diastolic filling from persistent SVT, the reduction in MAC, combined with potential antiarrhythmic properties, may support acepromazine use; however, high doses may be detrimental due to profound vasodilation and may perpetuate hypotension. In the present study, conditional inference forest analysis identified acepromazine (ACP) premedication and intra-operative paracetamol as possible predictors of hypotension. Subsequent analysis of these variables in logistic regression models failed to produce significance. As these tests were likely underpowered, the results are inconclusive. Hypotension secondary to α₁-adrenergic receptor-mediated vasodilation following ACP administration is well understood in veterinary medicine (31). However, an association between paracetamol administration and hypotension has not been reported in veterinary patients. In human medicine, intravenous paracetamol has been shown to decrease mean arterial pressure (MAP) by up to 15% and reduce cardiac output via activation of voltage-gated potassium channels on vascular smooth muscle (32, 33). To the best of the authors’ knowledge, this mechanism has not been investigated in the clinical veterinary literature and may be worth further investigation.

Fentanyl was administered to 24 patients in the present study, with 23 receiving a bolus and 3 requiring a constant rate infusion. The reason for administration was not clearly recorded on most anaesthetic records; the retrospective nature of this study meant that there was no standardised protocol for the use of fentanyl in these patients. Fentanyl, a short-acting pure *μ*-opioid receptor agonist, is frequently used to provide ‘rescue analgesia’ for breakthrough nociception during anaesthesia. However, its use in this cohort may have been related to its ability to reduce heart rate, which can facilitate termination of supraventricular tachycardias through enhancement of cardiac vagal tone (34, 35).

Due to the retrospective nature of the study, it remains uncertain whether fentanyl was administered for analgesia or as an intervention during tachycardic episodes to reduce heart rate. Although fentanyl is not a recognised treatment for SVT in humans, it has been shown to prolong RR intervals and atrioventricular (AV) nodal conduction, as well as AV nodal and ventricular refractory periods, suggesting a theoretical antiarrhythmic effect (36). However, no evidence currently supports the use of fentanyl for managing or terminating SVT in either veterinary or human patients, and this could warrant further investigation.

The absence of *α*2-adrenoreceptor agonist use in this study likely reflects two main considerations. First, α2 agonists can produce significant dose-dependent cardiovascular effects, including peripheral vasoconstriction, reflex bradycardia, increased LV end-diastolic pressure and myocardial workload, and reduced cardiac output (37); this may exacerbate haemodynamic instability in patients with impaired cardiac function. Second, α2 agonists can suppress sinus and atrioventricular nodal activity, atrial excitability, and automaticity (38), potentially hindering pathway identification and arrhythmia inducibility (39). As reliable SVT induction and mapping are essential for procedural success, avoiding agents that depress conduction is particularly important in the EP setting (40).

Evidence regarding the effects of α₂-agonists in humans undergoing EP studies or RFCA remains inconsistent. In paediatric patients, dexmedetomidine has been reported to depress sinoatrial and atrioventricular nodal activity at infusion rates as low as 0.7 μg kg^−1^ h^−1^ following a 1 μg kg^−1^ bolus (41). In contrast, a retrospective analysis found that the use of dexmedetomidine infusions caused no significant changes in electrophysiologic parameters but did reduce the inducibility of SVT in children undergoing RFCA (39). To the best of the authors’ knowledge, there is currently no published veterinary data evaluating the use of α₂ agonists in dogs undergoing EP and RFCA; therefore, no comment can be made on the suitability of this class of drug for the management of dogs undergoing these procedures.

In the present study, four dogs received atropine to induce SVT, and one dog received it post-ablation to evaluate ablation success. Post-ablation testing is performed to assess procedural efficacy by attempting to reinduce the previously documented arrhythmia. This is achieved either by high-rate endocardial pacing or by administering anticholinergic agents, such as atropine, to initiate the arrhythmia (40). The persistence or reappearance of SVT following RFCA suggests incomplete pathway ablation or the presence of multiple arrhythmogenic pathways or foci.

All patients received isoflurane carried in oxygen and air for the maintenance of anaesthesia. EP studies in adult humans generally do not require patients to be anaesthetised; however, general anaesthesia is commonly needed to facilitate EP/RFCA in paediatrics/children, uncooperative/anxious patients, or where ventilatory support is required. Isoflurane and sevoflurane are known to prolong ventricular repolarisation, and it has been suggested that this may reduce arrhythmia inducibility and delay successful EP mapping and ablation (42). In a study where anaesthetic maintenance with isoflurane was compared to propofol TIVA during RFCA in children with SVT, no significant differences in arrhythmia inducibility or procedure duration were found. However, atrioventricular nodal conduction was slower in the propofol group compared to the isoflurane group (43). One case report in a 4-year-old child reports persistent supraventricular tachycardia after sevoflurane administration, which only resolved following cessation of inhalation agent; however, the underlying mechanism was not determined (44). No comparative literature exists investigating maintenance agents for EP or RFCA in veterinary patients. Given the relative availability of both isoflurane and sevoflurane in the veterinary referral setting, further research could determine whether one agent provides a procedural or hemodynamic benefit during EP and RFCA.

The most documented intraoperative complication was arrhythmia, with tachyarrhythmias predominating. OAVRT occurred in 88.9% of cases, atrial fibrillation in 13.9%, ventricular tachycardia in 5.6%, and focal atrial tachycardia in 5.6%. Single cases of VF and accelerated idioventricular rhythm (AIVR) were also observed. The high incidence of tachyarrhythmias is unsurprising, as all dogs underwent EP study and ablation for previously documented, or strong clinical suspicion of supraventricular tachycardia. In this study, it is not possible to determine the significance of these arrhythmias in relation to intraoperative complications due to confounding factors, such as variation in structural heart disease and severity, anaesthetic protocol, breed variability/signalment, BCS, and anaesthetic duration.

Bradyarrhythmias were observed, with transient third-degree atrioventricular (AV) block occurring in two cases following pathway ablation. In both instances, the block was self-limiting and pacemaker implantation was not required. During RFCA, complete or transient first-, second-, or third-degree AV block can occur due to accidental AV nodal ablation when the accessory pathway is in close proximity to the AV node, or from temporary impairment of AV conduction secondary to ablation-induced thermal injury (45). In humans, cryogenic ablation has demonstrated comparable success rates to RF ablation with a lower incidence of AV block and improved patient comfort (24). To the best of the authors’ knowledge, cryoablation has not yet been reported in clinical veterinary cases.

Intraoperative hypotension occurred in 65.1% of cases, with 75% requiring intervention to maintain normotension. This incidence exceeds that reported by McMillan & Darcy (46). This higher incidence could be due to multiple factors such as reduced cardiac output from pre-existing disease (e.g., TICM), impaired diastolic filling during periods of tachycardia, and variability in anaesthetic protocols. Future studies employing standardised anaesthetic protocols with controlled drug combinations could be valuable to better predict the likelihood of intraoperative hypotension. Hypotension compromises tissue perfusion and oxygen delivery, predisposing to acute kidney injury (AKI) through renal hypoperfusion (47, 48). Only one patient developed postoperative azotaemia consistent with AKI as defined by Muñoz-Blanco et al. (49), which resolved within 72 h. Postoperative AKI may have been underreported, as renal parameters are not routinely monitored unless clinically indicated. Given that greater severity and duration of hypotension increase AKI risk (50), routine postoperative renal screening may be warranted. In this study, neither the severity nor duration of hypotensive events was recorded, which is a potential limitation.

Cardiopulmonary arrest occurred in four patients (9.1%), three intraoperatively and one postoperatively. One arrest occurred 9 h post-extubation, following a presumed successful ablation with no intraoperative complications. CPR was unsuccessful, and the patient died. The patient in question had marked changes on pre-procedural echocardiography, including severe global chamber enlargement and reduced ventricular contractility (left atrial and ventricular enlargement, LA: Ao 2.38, fractional shortening 22%), ascites, and low body condition score (2/9). The reason for the arrest was reported to be ventricular fibrillation by the attending cardiologist and was non-responsive to standard CPR and defibrillation protocols (51). One patient experienced CPA 50 min after induction due to sustained supraventricular tachycardia, which then progressed to arrest. Defibrillation resulted in transient ROSC, with sustained ROSC achieved following intravenous adrenaline (0.01 mg kg^−1^) administration. The exact rhythm that occurred during the arrest was unclear from the patient records. The procedure was subsequently aborted, and the patient was discharged. Successful ablation was performed 7 months later, with this procedure not being included in the dataset. One patient experienced a sudden bradycardia under anaesthesia followed by asystole 15 min after induction; ROSC was achieved, and the patient recovered uneventfully. The cause of the witnessed arrest is unknown. One patient went into CPA during post-ablation testing involving high-rate endocardial pacing; the dog developed ventricular fibrillation (VF) triggered by a ventricular premature complex (or premature ventricular complex, PVC) coinciding with a paced beat; a similar trigger of VF has been documented in humans (52). In this patient, repeated single shock electrical defibrillation was used as part of successful CPR. The European Resuscitation Council 2021 Adult Advanced Life Support guidelines, along with the veterinary RECOVER initiative (51, 53), recommend early electrical defibrillation in patients in CPA with a shockable rhythm (VF or PVT). These guidelines typically advise delivering a single shock after each 2-min cycle of BLS. However, in human medicine, a witnessed CPA with a shockable rhythm occurring during cardiac procedures is recognised as an exception (53). In such cases, “stacked shocks,” up to three sequential defibrillations delivered in rapid succession, are recommended prior to initiating chest compressions (53). Evidence from a porcine study demonstrated that the success rate of defibrillation (defined as termination of VF) increased from 44% with single shocks to 89% when three stacked shocks were used (54). This contrasts with current veterinary RECOVER guidelines, which do not recommend stacked shocks (51). Given that the arrest in this case was witnessed and likely iatrogenic, it is reasonable to hypothesise that the use of three stacked shocks before initiating BLS may have improved the outcome, despite the eventual success achieved using the standard protocol.

The incidence of cardiopulmonary arrest (CPA) during the intraoperative or immediate postoperative period in the present study population was 9.1%, with a median ASA physical status classification of 3. This rate of peri-operative arrest is higher than previously reported peri-anaesthetic mortality rates of 1.3–4% in dogs with comparable ASA status (55–57). Nevertheless, the overall mortality rate in the current study (2.3%) was consistent with the aforementioned reports for ASA 3 dogs. Due to the risk of CPA in these patients, standardised CPA management protocols, including explicit delegation of roles and responsibilities, should therefore be implemented during electrophysiological studies to optimise team performance and patient outcomes, regardless of ASA status. Simulated human CPR scenarios have shown that defined role allocation and leadership improve team communication and efficiency, potentially leading to better outcomes (58).

This study has several limitations. As a retrospective study, data depended on the completeness and accuracy of medical and anaesthetic records. Underreporting of complications, particularly transient or non-haemodynamically significant arrhythmias (including paroxysmal SVT, VPCs, bundle branch block or first/s degree AV block) may have occurred peri-operatively. Variability in anaesthetic protocols, parameter thresholds, and intraoperative interventions reflected individual anaesthetist preferences and may have introduced confounding factors. In addition, procedures were performed by anaesthetists with variable levels of experience in EP and RFCA, which may have influenced both the recognition and reporting of complications. Sample size, while suitable for descriptive purposes, was insufficient to detect statistically significant associations between management and complications. Prospective studies with standardised protocols and systematic intra and postoperative recording could be used to address these limitations in the future. For the given effect sizes and sample size, all the logistic regression models were underpowered. Non-significant results could be due to non-significance or type 2 error, making it difficult to draw any meaningful conclusions. There was no applicable sample size calculation as there was no specific hypothesis to test; rather, the data were evaluated opportunistically for any predictors. Including the logistic regression analysis may provide guidance for planning future studies.

An important limitation to consider is the variability in the severity of underlying cardiac disease within the study population. It is plausible that more advanced cardiac pathology may predispose patients to intraoperative haemodynamic instability, arrhythmias, and a greater need for intervention; however, this was not explicitly quantified or controlled for in the present study. Echocardiographic findings were classified as either ‘normal’ or ‘abnormal’. Abnormalities on echocardiography were not identified as a possible predictor for intraoperative complications by the conditional inference forest during statistical analysis. Further investigation quantifying disease severity and evaluating associated outcomes may help to determine whether a correlation exists between the severity of cardiac structural disease and complications/outcomes.

In conclusion, this study is the first to describe anaesthetic management and peri-operative complications in dogs undergoing general anaesthesia for electrophysiological studies and radiofrequency catheter ablation for treatment of supraventricular tachycardia. Anaesthetic protocols using a *μ*-opioid agonist with or without acepromazine, induction with propofol, and maintaining anaesthesia with isoflurane were adequate for procedural success. The most commonly encountered peri-operative complications included arrhythmias, hypoventilation, hypotension, and hypothermia, and tended to be mild, easily manageable, and resulted in no known lasting harm to patients. Nevertheless, catastrophic complications, including CPA and malignant arrhythmias, can occur throughout the peri-operative period, meaning clinicians must be prepared to manage these urgently; the mortality rate in dogs undergoing EP/RFCA in this study was 2.3%. When planning anaesthetic management in dogs presenting for EP/RFCA, we should tailor management to the needs of the individual, including considering the severity of underlying cardiac disease, with preference for agents that minimise haemodynamic and conduction interference.

## Data Availability

The original contributions presented in the study are included in the article/supplementary material, further inquiries can be directed to the corresponding author.
